# Wafer scale manufacturing of high precision micro-optical components through X-ray lithography yielding 1800 Gray Levels in a fingertip sized chip

**DOI:** 10.1038/s41598-022-06688-5

**Published:** 2022-02-17

**Authors:** S. M. P. Kalaiselvi, E. X. Tang, H. O. Moser, M. B. H. Breese, S. P. Turaga, H. Kasi, S. P. Heussler

**Affiliations:** 1 Singapore Synchrotron Light Source (SSLS), National University of Singapore (NUS), 5 Research Link, Singapore, 117603 Singapore; 2 Institute of Materials Research and Engineering, 2 Fusionopolis Way, Singapore, 138634 Singapore; 3 Attonics Systems Pte Ltd, 10 Anson Road, International Plaza, Singapore, 079903 Singapore; 4 Karlsruhe Institute of Technology (KIT), Institute of Microstructure Technology (IMT), Postfach 3640, 76021 Karlsruhe, Germany

**Keywords:** Energy science and technology, Engineering, Materials science, Nanoscience and technology, Optics and photonics

## Abstract

We present a novel x-ray lithography based micromanufacturing methodology that offers scalable manufacturing of high precision optical components. It is accomplished through simultaneous usage of multiple stencil masks made moveable with respect to one another through custom made micromotion stages. The range of spectral flux reaching the sample surface at the LiMiNT micro/nanomanufacturing facility of Singapore Synchrotron Light Source (SSLS) is about 2 keV to 10 keV, offering substantial photon energy to carry out deep x-ray lithography. In this energy range, x-rays penetrate through resist materials with only little scattering. The highly collimated rectangular beam architecture of the x-ray source enables a full 4″ wafer scale fabrication. Precise control of dose deposited offers determined chain scission in the polymer to required depth enabling 1800 discrete gray levels in a chip of area 20 mm^2^ and with more than 2000 within our reach. Due to its parallel processing capability, our methodology serves as a promising candidate to fabricate micro/nano components of optical quality on a large scale to cater for industrial requirements. Usage of these fine components in analytical devices such as spectrometers and multispectral imagers transforms their architecture and shrinks their size to pocket dimension. It also reduces their complexity and increases affordability while also expanding their application areas. Consequently, equipment based on these devices is made available and affordable for consumers and businesses expanding the horizon of analytical applications. Mass manufacturing is especially vital when these devices are to be sold in large quantities especially as components for original equipment manufacturers (OEM), which has also been demonstrated through our work. Furthermore, we also substantially improve the quality of the micro-components fabricated, 3D architecture generated, throughput, capability and availability for industrial application. Manufacturing 1800 Gray levels or more through other competing techniques is either limited due to multiple process steps involved or due to unacceptably long time required owing to their pencil beam architecture. Our manufacturing technique presented here overcomes both these shortcomings in terms of the maximum number of gray levels that can be generated, and the time required to generate the same.

## Introduction

There has been an active market demand for three-dimensional micro and nano components that require a large number N of gray levels for various applications in the last two decades pushing the requirement for such work^1–7^. Pencil beam-based lithography techniques such as e-beam lithography^8^ and proton beam lithography^9,10^ can yield precise components but take a longer time. Requirement for complex masks, resist shrinkage, and low throughput, limit the other patterning techniques such as direct-write gray scale lithography and two photon lithography to just research applications^2,11–13^. The LiMiNT x-ray beamline facility specially designed for micro/nanofabrication at SSLS^14–16^ offers a wide beam architecture that enables wafer scale fabrication. A number of work reports manufacturing high aspect ratio and highly precise components made using x-ray lithography^14,17–21^. However, to the best of our knowledge, fabrication of 1800 gray scales has not been demonstrated by any of the existing microfabrication techniques. Our work primarily involves usage of Synchrotron-based LIGA technique (a German acronym for Lithographie, Galvanik und Abformung) which translates into a three-step micromachining process involving lithography, electroplating and moulding. The process involves micro-patterning of polymers using x-rays, subsequently, electroplating the polymer mold and finally replicating using the generated metal mold^22^.

Using membrane based masks such as graphite membrane for x-ray lithography^23–26^, it is not possible to achieve micro-components of optical quality. In our work, we used stencil masks^27–29^, custom designed and manufactured to fabricate the novel architecture of micro-components required for various analytical devices. Conventionally used small area mask membranes such as SiO_2_, Si_3_N_4_ are fragile, hence, it is not suitable to generate large area masks that can enable wafer scale, despite having the preferred feature of lesser x-ray absorption. By large area mask, here we refer to a wafer of diameter 4″ and higher, applicable for large scale manufacturing to cater for industrial requirements. These masks are used a few 100 times before it is worn off or being accidentally damaged, hence have a requirement to be robust. SiO_2_ or Si_3_N_4_ membranes, might not survive such multiple handling especially as a large area mask. Stencil masks have gold structures as x-ray absorbers which are supported by relatively much rigid 100 µm thick Si frames, hence they are suitable for multiple usage for over few hundred exposures and multiple handling involved during the experimental process.

Hence, we used commercially available 4″ Si wafers that can serve as large area masks, as well as can be etched to create stencils. Lastly, through usage of single x-ray mask, it is not possible to achieve micro components with 1800 gray levels. Different stencil masks were specifically fabricated to generate the required unique architecture. Combinations of stencil masks were used simultaneously to realize formation of different segments of the 3D architecture.

Primary pattern required for x-ray masks are typically generated using UV lithography^30^. The diffraction effects that occur on the primary pattern due to UV rays are copied onto the fabricated microstructures. To overcome this, we performed an experimental study to compare the influence of different sources used to generate the primary pattern and its subsequent influence on the functional structure generated by x-ray lithography. This study also offers a solution to the influence of diffraction effect copied from the primary pattern generated in the x-ray masks to the functional structures. Another challenge in our process was the removal of polymer from the stencil masks which is dependent on the feature size^31,32^ which was also addressed through our work.

The conventional gold electroplating used to generate x-ray masks through continuous DC plating exhibits grain sizes large enough to lead to staggered side walls^33^. Typical high aspect ratio structures made using x-ray lithography^34–37^ which was the fundamental purpose of it, are not so sensitive to the roughness of the membrane or the grain size of the gold deposited as absorbers in the x-ray mask, since the photoresist apart from the functional structures is completely etched away. Hence, the finesse of etched surfaces was not of concern. In contrast, our work requires generation of 1800 gray levels. Since these micro-components later serve as functional optical components, the surface roughness of the etched surface and the finesse of side wall is of utmost significance. Rough sidewalls and edges of the components lead to unwanted scattering of the light that passes through it, inhibiting the functionality of the device. Importance of pulsed DC plating in our process was also studied. Various studies related to fabrication of customised stencil mask is available in supplementary section.

Our work, as presented in this paper, offers a solution to various challenges encountered in fabrication of N gray levels, through a synergy of techniques that are encompassed. The major advantage of our process is that 1800 or more gray levels can be achieved through single exposure run. Wafer scale fabrication has also offered the pathway to large scale manufacturing to cater for industrial requirement adding commercial usage to the developed methodology. The developed technique has been demonstrated on thin films on both Si and glass substrates, as well as on bulk polymers that are optically transparent and are of clinical grade. Hence, the manufactured components can directly serve as functional devices both with and without substrates. Metal replica of fabricated components on wafer scale have also been fabricated in collaboration with industrial partner , enabling large scale manufacturing through well established industrial techniques such as nanoimprinting^38^.

## Materials and methods

Methodology for fabrication of 3D micro-components of optical quality is discussed in this section.

### Substrate preparation

**Figure 1:**
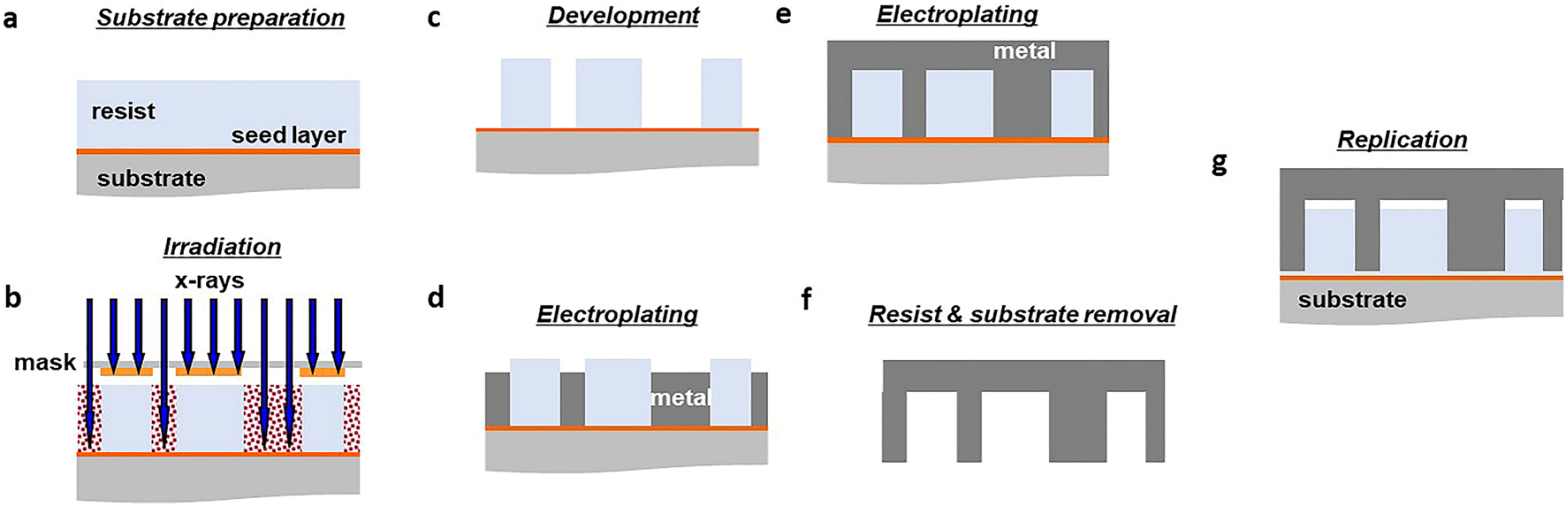
(**a**) Deposition of seed layer or metal layer on substrate for electroplating and thin film of photoresist for patterning using x-rays (**b**) Selective exposure of photoresist through x-ray masks (**c**) Development of photoresist where parts exposed to x-rays are removed and unexposed part remains (**d**) Electroplating of photoresist mold from metal seed layer (**e**) Completion of electroplating (**f**) Removal of photoresist and substrate to release the metal mold (**g**) Replication using the fabricated metal mold.

The micro-components are directly etched onto a commercially available bulk polymer sheet or on a thin film deposited on Si or glass substrate with a seed layer, as shown in Fig. 1a. We deal with positive resists throughout this paper. In case of using thin films, the substrate is firstly cleaned with acetone, IPA and DI water and then heat treated at 180 °C for about 10 min to remove any residual moisture. Substrate is then coated with a film of adhesion promoter to improve adhesion between polymer and substrate. Adhesion promoter used is Surpass, supplied by Teltec Semiconductor Pacific (Singapore) Pte Ltd. Followed by that, a polymer film of thickness 5 µm is spin coated using commercially available polymers such as poly (methyl methacrylate) (PMMA) with spin parameters chosen depending on the required thickness of the film. The thin film is then baked at 180 °C for 2 min to evaporate the solvent and then cooled down rapidly to room temperature. PMMA A11 of Kayaku make, procured from Teltec Semiconductor Pacific (Singapore) Pte Ltd, was used for this purpose.

### Irradiation

When a membrane-based single x-ray mask is used, the exposure is rather simple and straightforward with exposed part of the photoresist having their polymer chains destroyed or crosslinked, as shown in Fig. 1b. Since multiple stencil masks are used for exposure in our case, they need to be aligned with respect to one another, as well as with the sample^39^. Firstly, substrate and mask 1 are firmly fixed to the sample stage on top of the PI micro-controller^40^ through specially defined fixtures. The second mask is placed on top of mask and substrate assembly, using another fixture. The sample and first mask are moved together with respect to the second mask to complete the first set of exposure. Upon completion of it, the sample is removed from the exposure chamber and re-aligned once again with second mask for the second set of exposure and then irradiated. Details on the fabrication of stencil mask is provided in the supplementary section. Supplementary Fig. 1 shows line sketches and scanning electron microscope (SEM) images comparing the influence of UV copied and laser written stencil mask structures on the final microstructures fabricated. Supplementary table 1, compares the taper angle of microstructures in the stencil mask fabricated using direct laser writing and UV lithography.

In terms of distance between masks and sample, first stencil mask is placed in contact with the sample and the distance between the first and second stencil mask is about 1–2 mm. The spectral flux at the sample from Synchrotron covers a bandwidth from 2 to 10 keV (0.6–0.12 nm) and delivers a power of 0.9 W to a 4" wafer at a given electron current of 300 mA. Such shorter wavelength leads to very minimal diffraction effect.

This process is unique for 3D architecture that comprises of lamellae gratings and with bottom surface stepped in both directions, whereas, for micro-components with only stepping in x and y direction, the exposure is relatively simpler with single exposure run and single alignment. The whole setup including the sample, lamellae mask and steps mask, aligned on top of the movable stage, is loaded into the vacuum chamber of the x-ray scanner and evacuated. Fine movements of the masks and scanner stage are sequentially controlled, in micron-scale using software that controls sequentially the motion of the respective stages. Parameters such as velocity of movable stage, beam current, initial position of movable stage in both x and y directions, dosage for each exposure subjective of the etch depth, scan length and turnaround time are the other parameters that need to be determined prior to an exposure run^39^. The entire exposure is run automated via software control. The duration of x-ray exposure could vary based on the beam current and maximum target depth. For an etch depth of 150 μm and with a beam current of 240 mA at SSLS, the exposure time is roughly about 3 hours. This duration is also influenced by other parameters such as speed of micro-controller, scan distance, scan velocity and turnaround time. The dosage increments with respect to increasing etch depth is not linearly related, hence, prediction of exposure time is dependent on the etch depth according to a known numerical function. In the x-ray lithography process run, the major difference of our process from conventional process is the usage of multiple stencil masks to generate unique 3D architecture with 1800 gray levels or even more. In a wafer of 4″ diameter, about 40 chips per exposure could be accommodated when the chip size is (4.8 mm ×4.8 mm) and about 150 chips in case of a chip size of about (2.4 mm ×2.4 mm). The size of the chip is determined based on the size of the photodetectors on which it will be mounted later. The number of chips that can be manufactured in a single exposure run is in general restricted by the surface area of the wafer. It was also observed that when the grating structures that are commonly used as test structures were oriented parallel to the plane of the electron orbit of the synchrotron radiation source, the bottom surface of grating structures was tapered, whereas the bottom surface was flat when gratings were oriented perpendicular to the orbit plane. The likely cause is the difference between horizontal and vertical emittances of the electron beam in a storage ring, that the horizontal and vertical irradiation angles are about 20 and 1 mrad, respectively, when the sample is placed in an x-ray exposure station typically 10 m away from the electron orbit.

### Post exposure treatment

The exposed sample is unloaded from the scanner chamber and removed from the movable stage. It is then placed into the oven, heated to 90 °C and annealed overnight, as a means of stress release, structure and surface improvement, which is the typical purpose of annealing.

The annealing process however did not significantly improve roughness or surface profile in case of thin films deposited on a substrate, hence not applied. Upon completion of the annealing process, the oven is ramped down, and the sample is taken out for development. Overnight annealing also helps in removal of degassing by-products of the polymer used. It was observed that without post exposure annealing, it results in the formation of bubbles inside the sample due to trapping of gas resulting from polymer degas. Vapour of chloroform was also used in some cases to smoothen the surface roughness caused during etching of polymer layers that are deeper than 100 µm^41^.

### Development

In this step, typically exposed photoresist is removed in case of positive tone photoresist such as PMMA as shown in Fig. 1c. This is one of the crucial steps in the whole manufacturing process as different process parameters involved influence the etch depth and surface roughness achieved^42^. It needs to be optimized based on the period p (10, 20 or 40 μm) of the lamellae grating and the etch depth. However, the basic steps in the development process are development using GG developer, rinsing of the developed sample using GG rinse^43^ and rinse off of the residue on the sample using DI water. GG developer and GG rinse are chemicals prepared in-house using basic ingredients such diethylene glycol monobutyl ether (DGME), ethanolamine, morpholine and DI water. GG developer is mixed in a ratio of, DGME (60%), Morpholine (20%), Ethanolamine (5%) and DI water (15%). GG rinse is mixed in the ratio of DGME (80%) and DI water (20%). The chemicals are mixed in a glass beaker at 30 °C and continuously stirred at 200 rpm for uniform mixing and subsequently stored in amber glass bottles. Final cleaning of the sample with a stream of water at high pressure is done to remove the residues and sediments deposited at the surface of the sample, especially for structures with trenches deeper than 100 µm. For trenches less than 100 μm, the developer and rinse solution are continuously stirred at 200 rpm to assist the chemicals to enter deep trenches and remove any polymer residues there. Both high pressure DI water rinse and stirring are not required for polymer films that are less than 10 µm thick. It is also vital to place the samples facing down in the developer, which helps continuosly remove residues from the development process out of the narrow trenches.

### Metallization and replication

The developed samples represent the polymer mold, using which a metal mold can be formed through electroplating process, as shown in Fig. 1d,e. A conductive metal film called seed layer is deposited to enable electroplating. In our case, the polymer mold is not etched to the bottom, hence metal layer is deposited on top of polymer mold and electroplated from top surface. Upon completion of electroplating, the polymer mold is stripped off and the substrate is removed to release the metal mold as shown in Fig. 1f. The released metal mold can be used to create the replicas of the polymer mold, as shown in Fig. 1g. The replication process can be accomplished through a number of well-established industrial techniques including hot embossing and nanoimprinting.

## Results

A complement of techniques exhibiting synergy in achieving scalable manufacturing of high precision optical components is discussed in this section. Application of fabricated components to commercial analytical devices, comparison of our uniquely developed methodology to other peer techniques, and lastly, performance of analytical devices built using the micro components fabricated are also discussed.

### Aligned exposure using multiple masks

**Figure 2:**
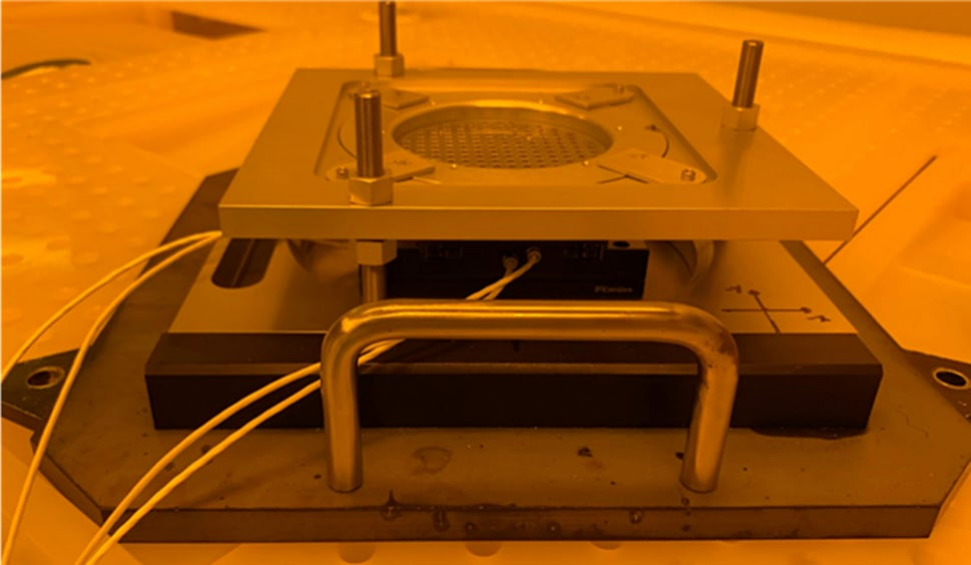
Fabricated aluminum fixture to align multiple stencil masks above the sample, whereby their relative position is controlled by a microcontroller.

The most important feature required to generate micro-components with surfaces of optical quality using x-ray lithography is the requirement of stencil masks. Typical membrane-based masks used in x-ray lithography such as graphite membranes when used, copy their surface roughness onto the components fabricated. Grating structures fabricated using graphite-based membrane mask exhibit surface roughness on etched planes^42^. On the contrary, when the same structure is fabricated using stencil masks that are free of membranes, the etched surfaces are of optical quality, with a surface roughness in the order of 10–50 nm. Dose distribution method used to achieve 1800 discrete gray levels is elaborated in the supplementary section including supplementary Figs. 2, 3, 4, 5, 6, and 7 comprising of colour maps of x-ray exposure dose, graphical and histogram plots. Statistical study was done on the surface roughness of 1800 gray levels and reported in the supplementary section including supplementary Figs. 8, 9, 10, and 11 which includes Whisker plots to evaluate the surface roughness values measured. Surface roughness values measured at equidistant cells across the entire chip with 1800 gray levels comprising in total roughness measurement of 98 points at 98 different cells is provided in supplementary table 2. Secondly, to generate unique 3D architecture developed through our work, simultaneous usage of more than one stencil mask is required^44^. Customised holder to assemble masks and substrate together with micromotion stages in it was designed and fabricated for this work, as shown in Fig. 2. 3D CAD drawing of the fabricated aluminium fixture is provided in supplementary Fig. 12. The micro movements of the stages are used to generate the unique 3D architecture, while the large movements of the sample holder were used to expose the area of an entire 4″ silicon wafer. The movement of micromotion stages is in the order of hundreds of microns, while the movement of the sample holder of x-ray scanner is up to 45 mm along vertical axis in both upward and downward directions. The stepped micro movements of the masks correlated with a precise control of dose deposited in each step offer determined (or “depth-controlled”) chain scission in the polymer in each step enabling 1800 discrete gray levels. For the structure with 1800 gray levels, one movable mask having a plurality of rectangular fields is scanned in two orthogonal directions. Additionally, a second static mask is placed on top of the photo-resist layer to confine the exposure field.

### Scalable manufacturing of the high precision optical components

At SSLS, x-rays emitted from circulating electron bunches reach the lithography station at a distance of more than 10 m from their point of origin. Characteristically for synchrotron radiation, horizontal and vertical acceptance angles of 20 and 1 mrad, respectively, produce a highly collimated, rectangular cross section beam enabling full wafer scale fabrication due to vertical scanning of the wafer.

**Figure 3:**
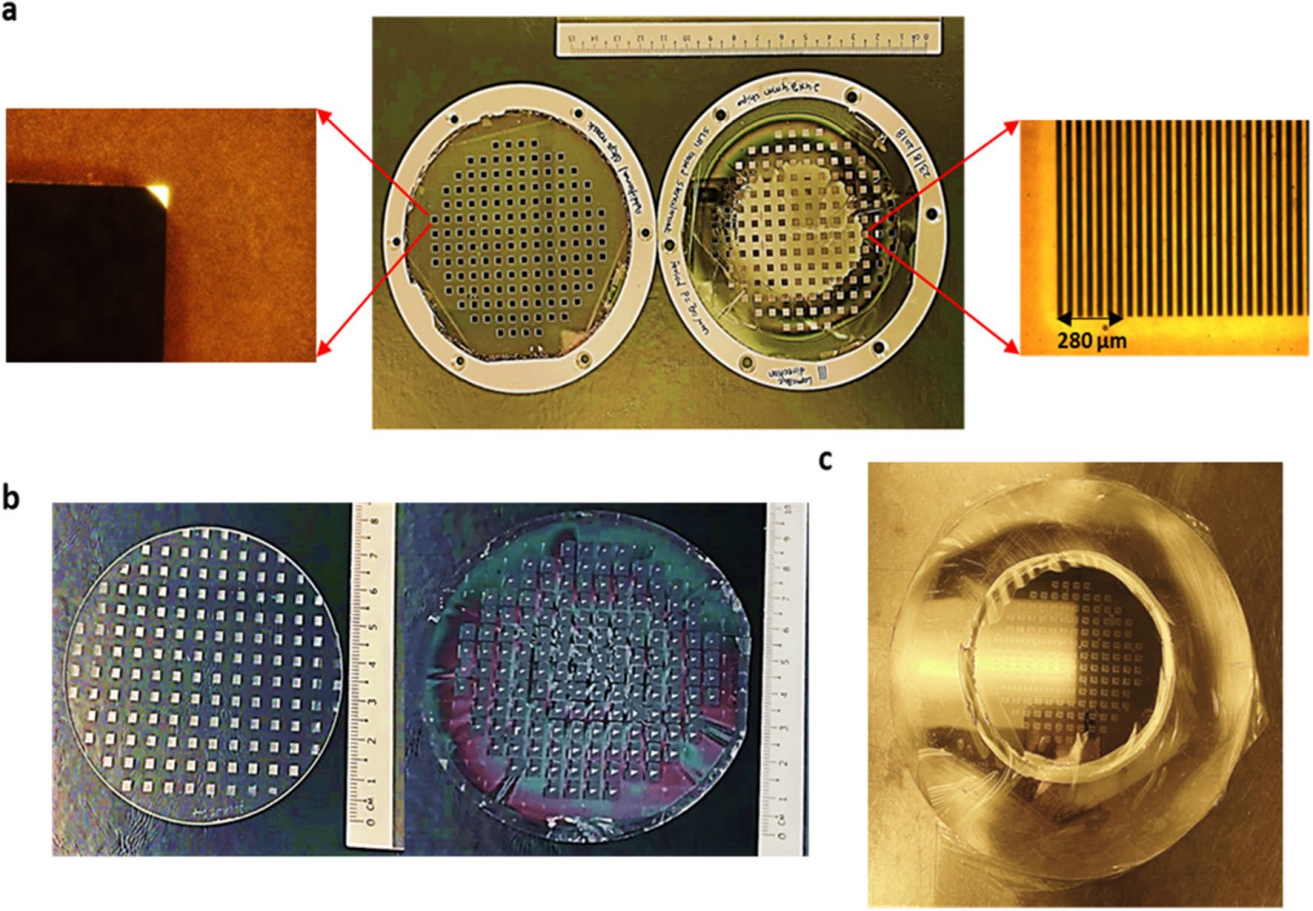
(**a**) Two different Stencil masks used to generate micro-components of 3D architecture, with zoom out of individual micro-chips acquired using optical microscope (**b**) Commercially available PMMA sheet of 80 mm diameter (left) and 4″ glass wafer (Right) completely exposed in single exposure run (**c**) Metal mold replica of the fabricated micro-components on wafer scale. View on the replicated area is somewhat obstructed due to its glossy surface reflecting the ceiling light.

Horizontally, the beam is confined by an aperture stop to about 10 cm width where it has a flat profile due to the sweeping electron beam. Vertically, the intensity distribution is approximately of a width of about 8 mm and the intensity is distributed in a Gaussian profile. Figure 3a shows images of a pair of stencil masks with their zoomed in images acquired using optical microscope, fabricated and used to generate a unique 3D architecture for specific application. Figure 3b shows a commercially available PMMA sheet of 80 mm diameter (left) and a 4″ glass wafer (right) exposed in single exposure run for parallel processing of multiple micro-chips. Using multiple stencil masks, it is possible to generate 3D stepped architecture on a wafer scale in a typical exposure duration of about 30–40 min. Given such a short time, it is possible, at SSLS, to accommodate 5–10 exposures in a day just during the day shift of synchrotron run. This translates to patterning of roughly 750 to 1500 micro-chips in a day. Precise metal mold replica of this sample was fabricated as shown in Fig. 3c and applied to industrial replication techniques such as nano imprinting, offering the potential to increase the quantity manufactured in a day by one to two orders of magnitude.

**Figure 4:**
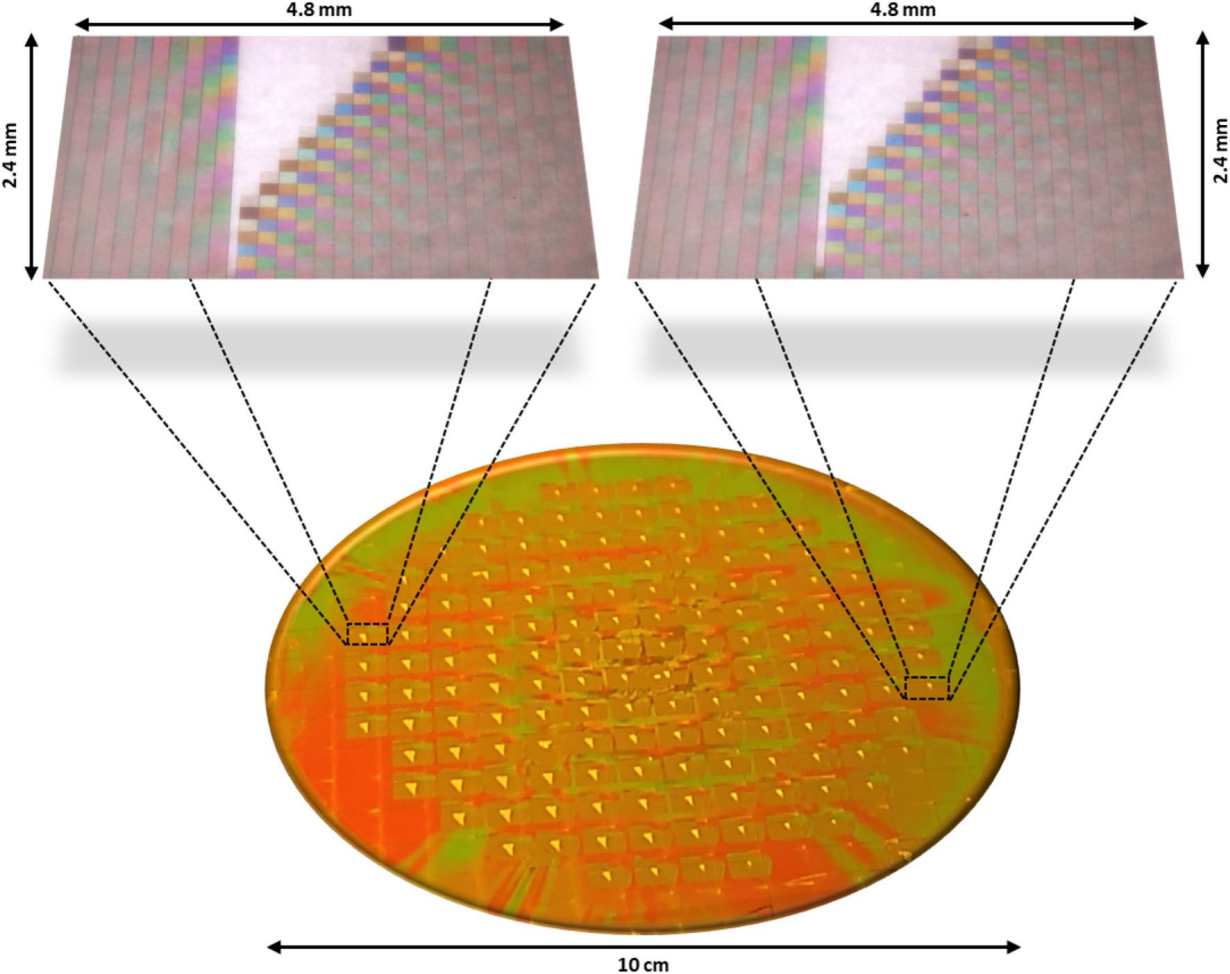
Image of wafer scale fabrication of microchips with 148 chips in wafer achieved through single exposure run and the zoom out of individual chips at the left and right end of the wafer. Zoom out shows the 1800 steps with varying height in both x and y direction. The blank triangular area at the center of the chip is the substrate area without any steps or any polymer and are used as a zero step for I_zero_ measurement of average power of unmodulated light that is incident on the substrate.

LIGA based x-ray lithography thus serves to fabricate micro/nano components of optical quality on a large scale for industrial requirement. Currently, our work offers single micro-chip sized 5 × 4 mm^2^, comprising about 1800 unique phase arrays or optical filters. About 150 such micro-chips are created in parallel on a 4″ silicon or glass wafer through single x-ray exposure run as shown in Fig. 4. Replication using the metal mold and through well-established industrial techniques such as nano imprinting has also been demonstrated.

### Shrinkage of architecture of analytical devices

**Figure 5:**
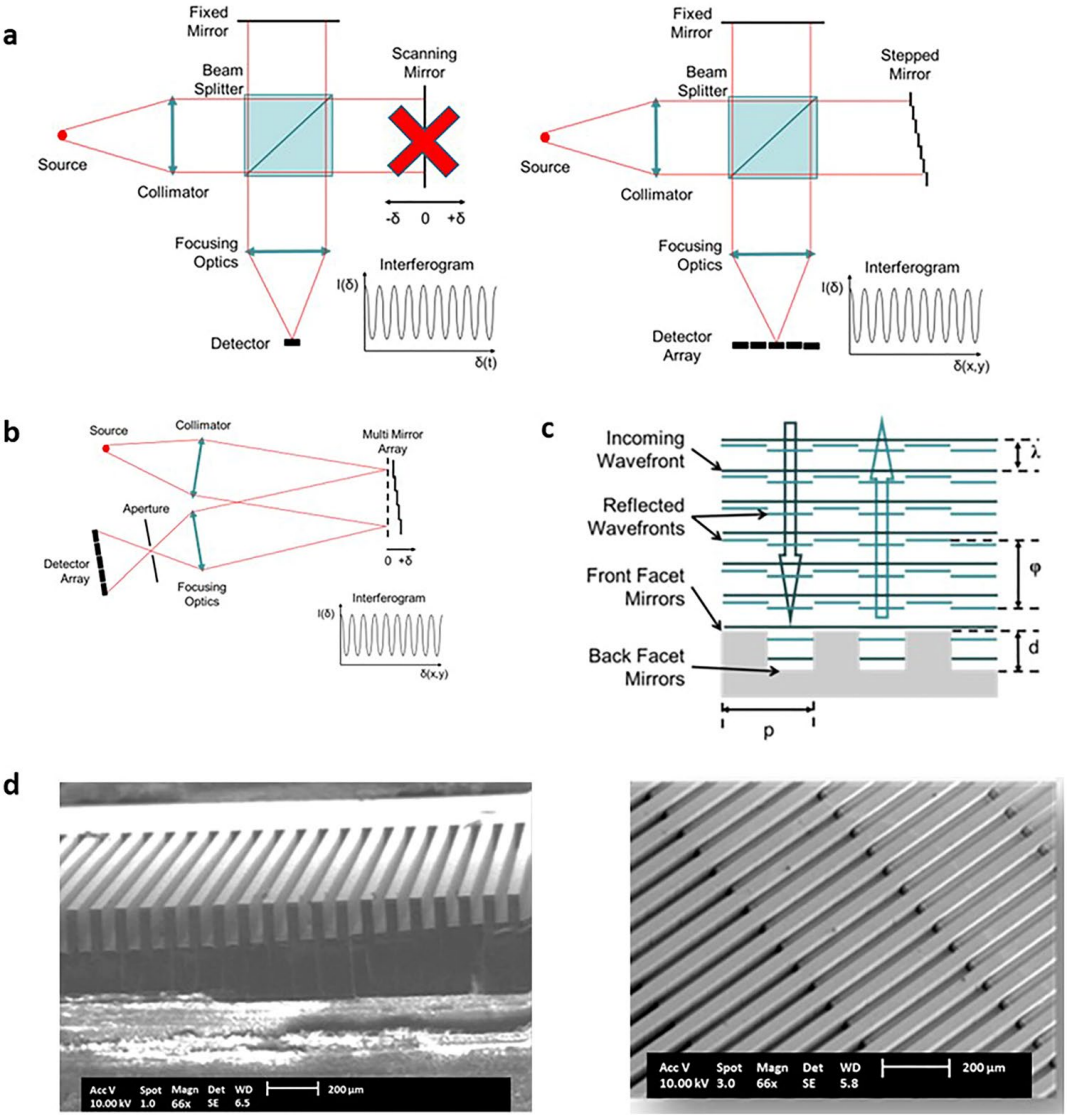
(**a**) Line sketch depicting the replacement of scanning optics with static optics in the form of stepped mirror in Michelson interferometer (**b**) Line sketch showing the acquisition of the signal from filter array by a detector array (**c**) Line sketch showing the fabricated micro-component functioning as wavefront beam splitter (**d**) SEM image of micro-components that functions as wavefront beam splitter.

Considering the Michelson interferometer that is quite common in FTIR (Fourier transform infrared interferometry) as an example, we show that the micro-chips fabricated using our unique methodology can replace the scanning optics with static optics as illustrated in Fig. 5a, making the spectrometer robust, as well as shrinking its size and weight. The signal from filter array is transferred to a detector array by imaging such as to maintain the unique correlation of phase difference on the interferometer chip with the position on the detector pixel array Fig. 5b. These micro-chips can either function as an amplitude beam splitter such as Fabry–Perot arrays or wavefront beam splitters^39,45^ as shown in Fig. 5c based on the design of the 3D architecture, customized and designed based on requirement. Figure 5d shows the SEM images of the 3D architecture of wavefront beam splitter manufactured using our methodology. It can be seen from the images that the micro-chip encompasses lamellae gratings and steps at the bottom of the gratings with their depth varying in both x and y directions. The top surface of the lamellae takes the role of the fixed mirror of a Michelson whereas the various steps at the bottom of the lamellae represent the various positions of the scanning mirror. Such complex architecture is impossible to be fabricated using the conventional single mask approach and through standard x-ray lithography process.

**Figure 6:**
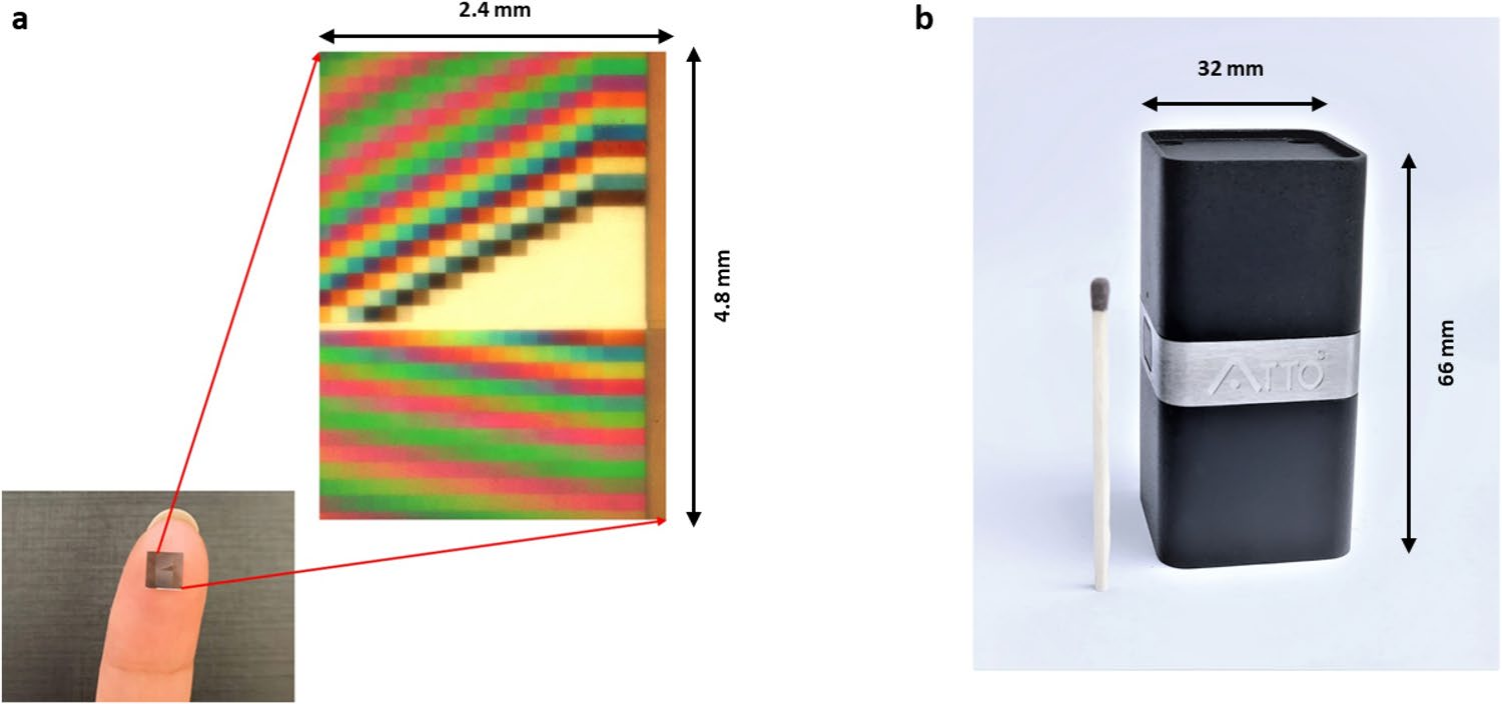
(**a**) the image of the micro-chip held in a fingertip exhibiting its minuteness and with its magnified version acquired using optical microscope. The magnified image shows an array of 1800 filters enclosed in a chip of size 4.8 × 2.4 mm (**b**) Picture of the spectrometer whose size shrank to handheld (66 × 32 × 32) mm^3^, due to the usage of micro-chips fabricated through the manufacturing methodology presented in this paper, drastically reducing the complexity and size of the device.

Figure 6a shows the image of the micro-chip held in a fingertip exhibiting its minuteness and with its magnified version acquired using optical microscope. The magnified image shows an array of 1800 filters enclosed in a chip of size 4.8 × 2.4 mm^2^. Each of the filters in the filter array acts as a discrete filter due to their unique gray level. The size of tabletop spectrometer shrank to handheld as shown in Fig. 6b due to the usage of fine micro-components manufactured using our presented work.

### Comparison of other microfabrication techniques

To manufacture 1800 gray levels, few of the currently available peer techniques are direct-write grayscale lithography and 3D laser structuring^2,11–13,29^. In 3D laser structuring, the number of gray levels that can be manufactured is not limited, however it takes prohibitively longer time due to its pencil beam architecture. Gray scale lithography in comparison does not suffer from this drawback and can generate micro-components in a 4- or 6-inch silicon wafer in a single exposure run. However, multiple process steps involved make the entire process very tedious and time-consuming, thereby limiting the number of gray scales that can be generated. An experimental comparison study was conducted to compare the fabrication time and the capabilities of other peer techniques currently available. It was seen that the time taken by those techniques to fabricate 150 chips of area 4 × 5 mm^2^ with about 1800 mirror arrays each is about two to four orders of magnitude higher than that required by LIGA based x-ray lithography. This is mainly because of the pencil beam architecture of the source used to write the samples. For the same reason, it is also not possible to perform batch fabrication using these methodologies.

**Figure 7:**
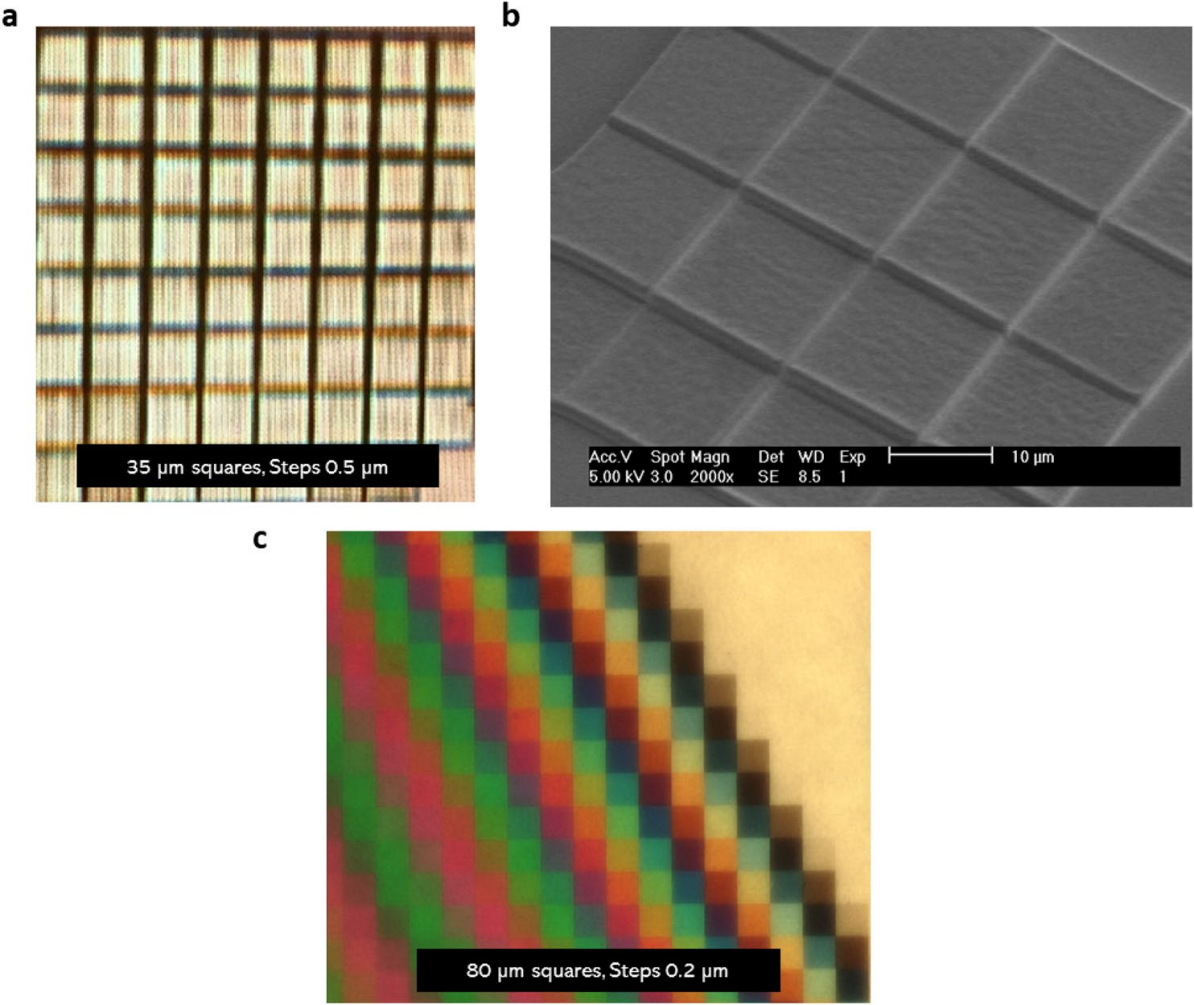
(**a**) Optical microscope image of microstructure written using commercially available 3D printing technique showing write lines making it unsuitable to serve as an optical component (**b**) SEM image of microstructure written using 2-photon lithography showing uneven surface making it unsuitable to serve as optical component (**c**) Optical microscope image of microstructure written using our novel manufacturing technique with surface roughness equivalent to an optical component, reflecting a monochrome color per square indicating uniformity and surface smoothness across entire square.

The novel technique developed by us overcomes both these shortcomings due to rectangular beam architecture that can expose entire 4″ wafer in a single exposure run and due to our unique methodology, that can generate N gray levels. Figure 7a shows the microstructure written using commercially available 3D printing technique^46^. The fabricated micro-component shows obvious write lines on the surface of the structure making it unsuitable to serve as an optical component. Figure 7b shows SEM image of microstructure written using 2-photon lithography, as well showing uneven surface making it unsuitable to serve as an optical component. Figure 7c shows microscopic image of the microstructure written using our novel manufacturing technique with surface roughness suitable to serve as an optical component. Comparison of surface quality of micro-components manufactured confirms that the surface quality offered through our technique is far superior for it to serve as an optical component. Each square of an area 80 µm^2^ in the micro-chip, reflects a unique monochrome color, indicating uniformity and surface smoothness across entire square.

### Performance of an analytical device built using the micro component fabricated

In this section, we present an application demonstrated by a handheld spectrometer, that uses the micro-chips fabricated through the manufacturing process presented in this paper. We demonstrate here a static interferometric based optical sensor capable of resolving fine atomic emission lines generated in a plasma. We showcase the monitoring of plasma cleaning of polymer layer from a silicon wafer and demonstrate the sensor capability to monitor gas mixtures in the plasma that occur during Reactive Ion Etching (RIE) of polymer thin film deposited on Si wafer. Plasma processes are of major importance in modern manufacturing. In a plasma, gas atoms are ionized and excited to higher energy states by external sources such as electric fields or flames, to name a couple. These ionized gas clouds find use in various industries including the semiconductor, medical or food industry in which either thin films are deposited/etched, or surfaces are altered for sterilization by tailoring the surface chemistry of packaging material from hydrophilic to hydrophobic through a plasma treatment. As an industrial standard for any process, accurate control over plasma-based processes is also of paramount importance.

**Figure 8:**
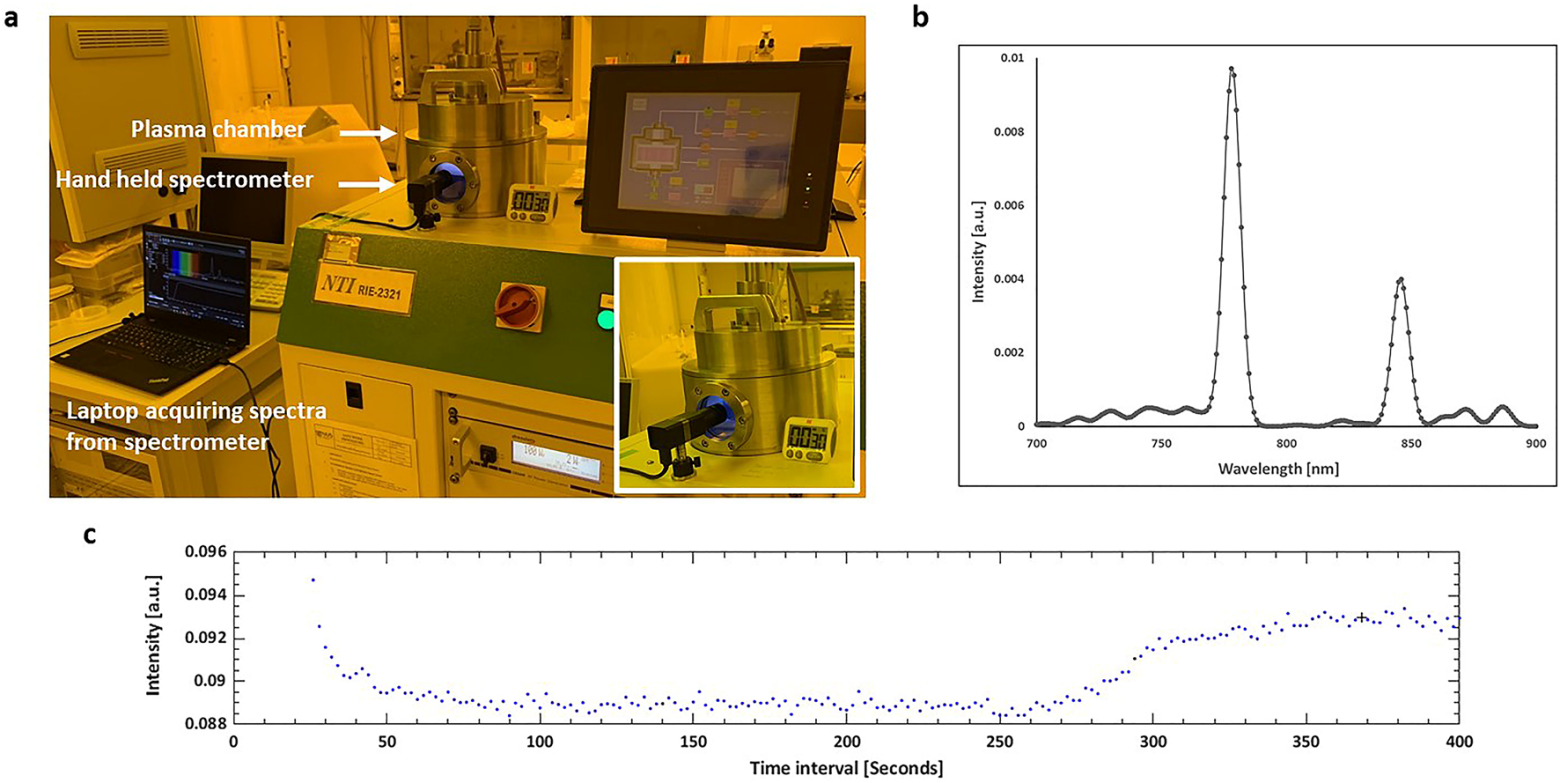
(**a**) Experimental setup with handheld spectrometer mounted onto the viewing port of RIE system and the spectra are acquired using laptop connected to the spectrometer. The insert within the picture shows the image of the spectrometer recording the emission spectra through the viewing port of plasma chamber (**b**) Plasma emission spectra of clean oxygen at a gas flow of 20 SCCM gas flowrate at a pressure of 150 mTorr and 100 W RF power. (**c**) Time-series recording of variation of intensity of oxygen emission line at 776 nm plasma emission during the etch of PMMA photoresist at 100 W RF power.

The VIS–NIR handheld spectrometer was attached to the optical viewing port of a plasma cleaner (NTI RIE-2321 Reactive Ion Etching System) as shown in Fig. 8a. The spectrometer recorded an emission spectrum showcasing its capability to monitor plasma process online, as shown in Fig. 8b, measuring the peak intensity of oxygen emission line at 776 nm. A 4-inch diameter silicon wafer was spin coated with PMMA photoresist (MicroChem A11). Then, we employed the plasma cleaner with oxygen plasma at 100 W RF power to remove the photoresist while monitoring the oxygen emission line over time. Flow rate of oxygen gas was maintained at 20 SCCM for a total plasma duration of 400 s and a spectrum was captured every 4 s. The data was collected by the handheld spectrometer following the etching of polymer layer on a silicon wafer. To follow the etching process in real-time, the emission of an oxygen line over the duration of the etching process was monitored. Figure 8c presents variation of area under curve of peak at 776 nm over time. The plasma was ignited after 30 s and thereafter maintained for a total duration of 400 s. The emission of oxygen at plasma ignition spikes up and decays during about 50 s to a low stationary level until about 260 s when it rises again to the initial level. The low stationary level indicates the phase when the resist is etched away. Once the polymer layer is removed completely, the oxygen emission increases again as the relative concentration of pure oxygen in the plasma increases. This experiment is a confirmation of the capability of the spectrometer equipped with micro-chip fabricated by the unique methodology proposed in this paper, to monitor plasma-based processes.

For applications in typical Fourier transform infrared (FTIR) system, the maximum depth of the chip determines the resolution whereas the minimum stepping of the phase steps (gray levels) determine the minimal wavelength to be resolved and thus the spectral bandwidth. The manufactured micro-components are currently used in the UV and Visible spectrometers, multispectral imagers and as light filters. Configurations required for far infrared region for a multichannel Fourier transform infrared (MC-FTIR) device are different and have already been demonstrated^45^. For micro-chip needed for the spectrometer to operate in mid-infrared region, the depth required is in the range of 500 microns, in which case bulk PMMAs commercially available as sheets are used, instead of thin films on substrates. For resonant structures, in comparison, the cell thickness and its stepping (through the gray scale process) determine the transmission peak wavelength position and wavelength shift per gray scale point. For spectral recovery methods, large number of cells is advantageous (in particular when considering detector noise) for noise suppression^47^.

## Conclusion

In summary, we have developed a robust methodology for the microfabrication of three-dimensional micro-components of optical quality in a scalable manner that can cater for industrial applications. In conjunction with well-established industrial techniques such as nano imprinting, we also demonstrated that such fine components can be replicated in the form of a metal mold. Such a metal mold can be used to increase the manufacturing capability by one to two orders of magnitude. Such large quantities are required when these micro-chips are to be incorporated into commercial devices or sold as OEM devices to meet consumer demands.

The presented results are not achievable through any of the existing micro-fabrication techniques especially at the given time. We had developed this technique over the past 16 years using a synergy of various techniques and benefitting from our multiple stencil mask technique, specifically developed for this process. Our results are compelling, as our manufactured components have already been applied in commercial products and are successfully sold in the market. Techniques to generate N gray levels are tried by many universities and industries. The methodology presented can be applied for fabrication of micro-components of optical quality for several applications potentially beyond the application for analytical devices discussed in this manuscript. Although our structures presented in this work are not arbitrary free forms, they vary in shape in all three directions x, y, z and are more than extruded shapes. This technique is well suited for chess board like patterns structured from the top surface of the photo resist.

Although not directly serving as a work horse for industries, synchrotron can serve as a foundation for generation of high precision micro-components on a wafer scale which can then be replicated and applied to nanoimprinting to cater for industrial needs.

## Supplementary Information

Supplementary Information 1.

Supplementary Information 2.

Supplementary Information 3.

Supplementary Information 4.

Supplementary Information 5.

## Data Availability

All data needed to evaluate the conclusions in the paper are present in the paper. Additional data related to this paper may be requested from the authors.
