# Supplementary material for: Wafer scale manufacturing of high precision micro-optical components through X-ray lithography yielding 1800 Gray Levels in a fingertip sized chip
Source: Sci Rep. 2022 Feb 17;12:2730. doi: 10.1038/s41598-022-06688-5 (PMC8854699; doi:10.1038/s41598-022-06688-5)
Supplement: Supplementary file 1 — Supplementary Information 1. [file 41598_2022_6688_MOESM1_ESM.pdf]

Fabrication of customised stencil mask

Stencil mask fabrication process has long been established[1, 2, 3] and pursued. However, the unique design and wafer scale mask of 4" diameter required for our process, had its own challenges, due to their fragility, especially after etching Silicon (Si) thickness off from its original 300 - 500 µm thickness down to 100 µm in the parts of wafer surrounding the functional structures. Collapsing of up to 4 mm long and 5 to 40 µm wide strings of lamellae structure was also one of the critical issues to be addressed. Furthermore, optimization was also needed for UV lithography parameters to transfer patterns from optical mask to stencil mask, due to the diffraction effects arising from the process. While several such issues were addressed through our work, we discuss here only the challenges encountered in generating microstructures with straight sidewalls in the stencil mask. Tapered profile of the microstructures in the stencil mask must be minimized as they are subsequently copied to the final microstructures fabricated, which greatly affects their functionality.

The typical process steps involved in stencil mask fabrication are mask design using Autocad software, primary pattern generation on optical mask blanks using laser writer (SUSS MA6 Mask aligner), Si wafer pre-treatment, thin film formation using SU8, a negative tone photoresist, on Si wafer, UV exposure, development, electroplating of gold absorber patterns, photoresist removal, and, finally, Si etching from backside of wafer to create Si stencils. Si wafer is the substrate used for the fabrication of stencil mask, which also acts as the supporting frame for the stencil. From our experimental studies, it was observed that when primary pattern from optical mask is copied onto the stencil/x-ray masks through UV lithography, the diffraction effects were prominent on the structure generated, as shown in supplementary Fig 1(a). It leads to tapered sidewall of mask features which was then copied to the final microstructures during x-ray lithography process as shown in SEM image in supplementary Fig 1(b). The degree of taper, alpha, is dependent on the difference between top and bottom width of the structure generated.

As an alternate approach, to avoid diffraction effects of UV lithography process, we generated the primary pattern directly on the stencil masks using laser (DWL 66 laser writer, Heidelberg Instruments). Dose variation was done on a trial-and-error basis until a satisfactory shape of the pattern, lamellae/grating in our case, was obtained in the stencil mask. The top and bottom width of the lamellae structures were measured in the stencil mask. These microstructures were generated using optimized laser parameters (Ar-ion at 362 nm, laser energy of 75 mW/cm^2 and 0 defocus length using 10 mm write head) and using UV source (i-line at 365 nm). It was seen that the width of the lamellae structure was same at the top and bottom when laser writing was the source of stencil mask pattern generation. Whereas the lamellae structures written using UV lithography for primary pattern generation, on an average had a difference in width of 2 µm between top and bottom. Supplementary table 1, indicates the taper angle alpha obtained for structures written using the two different methods used in this work to generate microstructures in the mask. Grating structures with different periods such as 20 µm (10 µm line and 10 µm space), 30 µm (15 µm line and 15 µm space) and 40 µm (20 µm line and 20 µm space) was used for this study.

**Method**	**Period 20**<br>**Aspect ratio = 1.4**	**Period 30**<br>**Aspect ratio = 2.14**	**Period 40**<br>**Aspect ratio = 2.86**
UV (365 nm)	Alpha > 5	Alpha > 5	Alpha < 5
	Not acceptable	Not acceptable	Acceptable
Laser (362 nm)	Alpha < 5	Alpha < 5	Alpha < 5
	Acceptable	Acceptable	Acceptable

Supplementary table 1. Comparison of taper angle of microstructures in the stencil mask fabricated using direct laser writing and UV lithography.

Comparing the stencil mask patterns generated using UV lithography and direct laser writing, we could conclude that for lamellae periods lesser than 30 µm, laser is suitable, as it yields straight sidewall, as shown in supplementary Fig 1(c). For lamellae periods greater than 30 µm, both laser and UV lithography are suitable as degree of slant of sidewall is relatively much lower. Based on the results obtained, it appears that diffraction effect caused by the collimated exposure field of UV lithography system, limits it's usage to periods greater than 30.

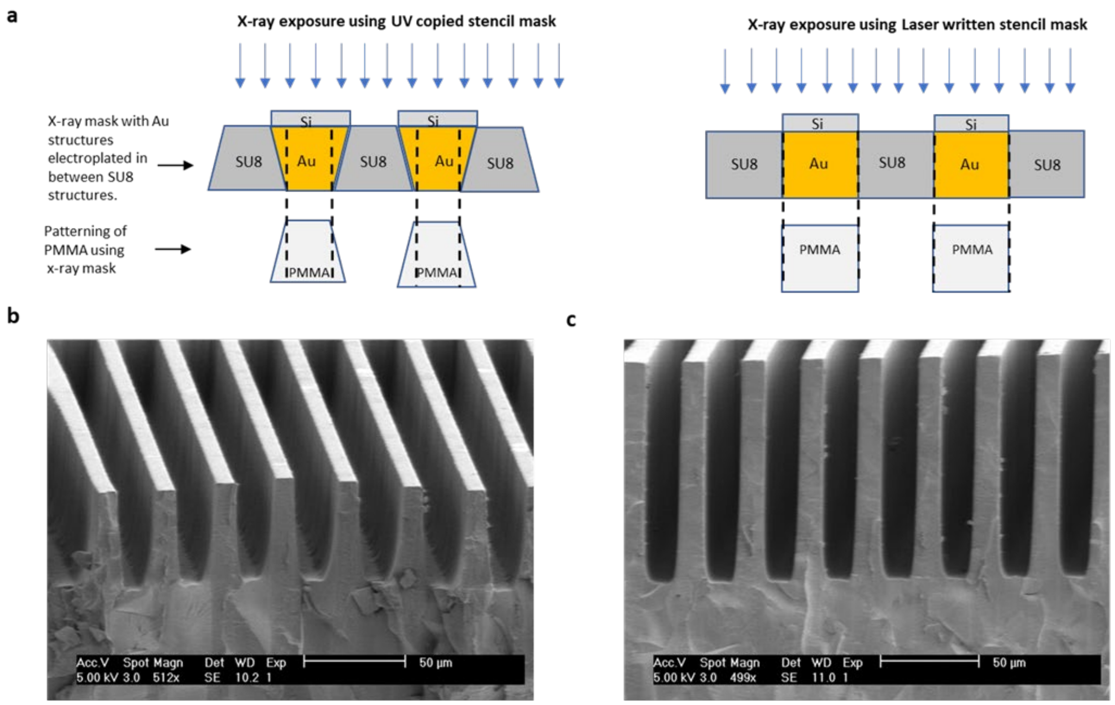

Supplementary figure 1. (a) Line sketch showing X-ray exposure using UV copied stencil mask leading to tapered sidewalls of functional structures of PMMA and X-ray exposure using laser written stencil mask leading to straight sidewalls of functional structures of PMMA (b) SEM image of grating structure fabricated using UV copied stencil mask leading to tapered sidewalls of PMMA (c) SEM image of grating structure fabricated using laser written stencil mask overcoming the problem of tapered sidewall and leading to straight sidewalls of the fabricated microstructure.

Removal of polymer from the stencil masks was another inherent challenge, especially when the space between gratings is less than 20 µm wide and greater than 50 µm deep. A combination of wet etching, ashing and plasma etching was carried out for complete removal of SU8. Our experimental studies showed that even a tiny residue of polymer material, stringing along open areas of stencil masks copied their impression to the final microstructure, affecting their functionality. Hence, it is vital to ensure complete removal of polymer before the Si layer underneath is etched away to create stencils. The equipment used for ashing of SU8 is Oven furnace of Labotherm make, model L5/11/C6, 5 Litres, purchased from Sino Chemical Co., Ltd., Singapore. When ashing was performed at 900°C, just in a hours' time, SU8 structures were burnt to ashes, however, the gold seed layer on top of the Si wafer as well as gold absorber structures lost its brightness as well as their adhesion to the wafer. At temperature of 400°C and below, SU8 structures could not be burnt to ashes. Hence, the optimized temperature that could ash SU8 structures without affecting gold structure was found to be 500°C in a time duration of 5 hours.

Gold is the commonly used absorber material in x-ray masks. The grain size of the gold particles, thereby the smoothness of gold structures fabricated is dependent on a number of parameters such as pH, temperature of the gold electroplating bath and the plating current density[4, 5]. Gold particles with larger grain size results in coarse/staggered side walls in the patterns in stencil-based x-ray mask which is then copied to the final microstructures fabricated. Hence, for our work we adopted pulsed electroplating which contributed significantly to obtain finer microstructures of optical quality due to finer grain size of gold particles. Gold electroplating requires electric current supply which can be done either in the form of continuous DC or pulsed DC. In continuous DC the gold components are continuously deposited, while in pulsed form, depending on the duty cycle given, gold ions are deposited in cycles. During pulse ON, Au ions are deposited on the substrate and during pulse OFF, the depleted ions are replenished in the vicinity of the surface to be plated for the subsequent pulse ON. Current density of the gold electroplating bath was 0.2 A/dm^2, at a pulse duration of 20 ms ON and 80 ms OFF and the type of electrolyte used was Enthone, procured from Cookson electronics, Singapore. It has been shown experimentally [see for example[6]] that the deposited gold film in pulsed electroplating is relatively more uniform exhibiting a finer grain size and thereby smoother surface finish of electroplated gold structures. Hence, the finer grain size results in generation of finer mask features, thereby finer microstructures with smoother sidewalls. It was crucial for our work that the edges of the gold absorber structures in mask is smoother to generate smoother sidewalls of the final microstructures.

References:

1. Mekaru, H., Takano, T., Awazu, K., Maeda, R. Fabrication of a Si stencil mask for the X-ray lithography using a dry etching technique. Journal of Physics: Conference Series 34, 1 859-864 (2006).
2. Du, K., Ding, J. J., Liu, Y. Y., Wathuthanthri, I., Choi, C. H. Stencil Lithography for Scalable Micro- and Nanomanufacturing. Micromachines 8, 4 24 (2017).
3. Yun, H. Y., et al. Stencil Nano Lithography Based on a Nanoscale Polymer Shadow Mask: Towards Organic Nanoelectronics. Nature scientific reports 5, 1 8 (2015).
4. Dauksher, W. J., Resnick, D. J., Johnson, W. A., Yanof, A. W. A new operating regime for electroplating the gold absorber on x-ray masks. Microelectronic Engineering 23, (1-4) 235-238 (1994).
5. Chiu, S. L., Acosta, R. E. Electrodeposition of low stress gold for x‐ray mask. Journal of Vacuum Science & Technology B: Microelectronics Processing and Phenomena 8, 6 1589-1594 (1990).
6. Ali, K., et al. Synthesis and Performance Evaluation of Pulse Electrodeposited Ni-AlN Nanocomposite Coatings. *International Journal of electrochemical science* **3**, 13 (2018).
