# Supplementary material for: Wafer scale manufacturing of high precision micro-optical components through X-ray lithography yielding 1800 Gray Levels in a fingertip sized chip
Source: Sci Rep. 2022 Feb 17;12:2730. doi: 10.1038/s41598-022-06688-5 (PMC8854699; doi:10.1038/s41598-022-06688-5)
Supplement: Supplementary file 2 — Supplementary Information 2. [file 41598_2022_6688_MOESM2_ESM.pdf]

Dose distribution method used to achieve 1800 discrete gray levels

To achieve 1800 discrete gray levels, doses can be deposited through three options.

1. By individually depositing discrete doses in each of 1800 gray levels, discrete gray levels can be achieved. However, this is very tedious and time-consuming; hence this option was not adopted. We do assume this option, however, for fewer gray levels, for instance, when just 10 discrete gray levels are required.
2. Another option is depositing constant dose steps through horizontal and vertical movements of the micromotion stage in a chess board like pattern. However, this option has a drawback: some gray levels are repeated due to the non-linear relation between dose deposited and etch depth.
3. Hence, we adopted option 2, but with variable dose in rows and columns. To achieve the same, we did the following,

The dose map shown in supplementary figure 2 (30 x 30 matrix on each side of the rectangular chip) was obtained using the constant dose strategy (option 2) to microfabricate the chess board-like pattern.

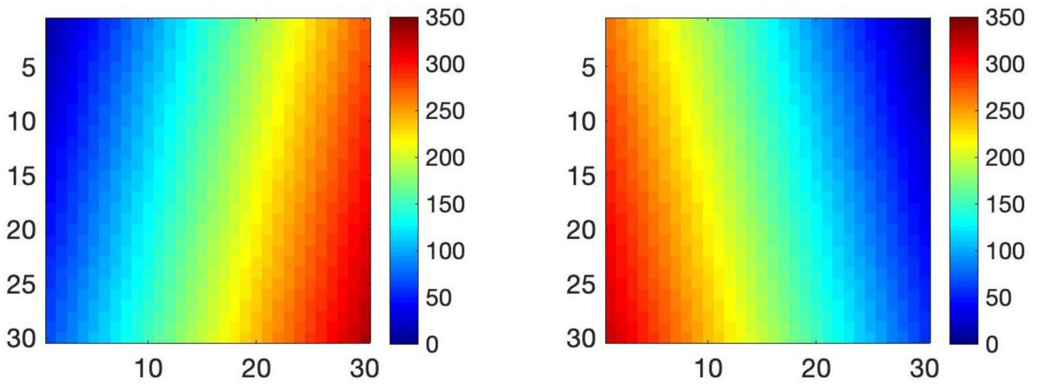

Supplementary figure 2. Colour map of x-ray exposure dose across the chip in constant dose deposition strategy (Red – High exposure and blue – low exposure). X and Y axis denotes the row and column numbers respectively.

Using the measured (cell transmission versus wavelength) from the microfabricated chip and fitting them using Fabry-Perot equations, each cell's depth/thickness can be estimated. From this estimation, it was realized that the cells did not have discrete cell depths and hence did not result in 1800 discrete gray levels.

To obtain discrete gray levels, we chose the unique cell depths from the data in the graphical plot in supplementary figure 3 and calculated the corresponding dose energy.

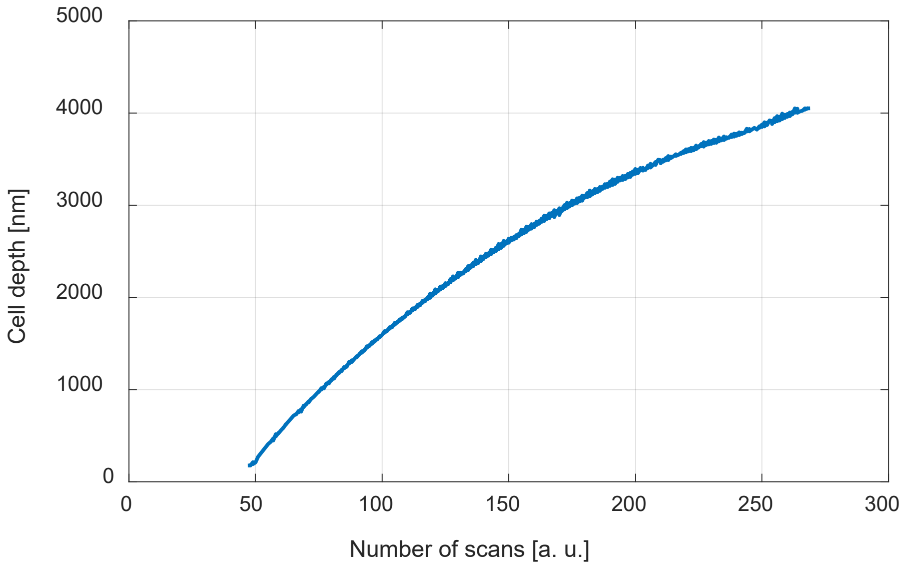

Supplementary figure 3. Graphical plot showing cell depths obtained for different scan numbers based on the constant dose strategy.

For computing the dose energy from the number of scans, equation 1 was used:

$$Exposure\ dose\ (J/cm^3) = \frac{Number\ of\ scans * Beam\ current}{Velocity\ of\ scans * 60} \quad (1)$$

We then fit a spline curve to energy dosage based on constant dose strategy to obtain unique cell depths, as shown in supplementary figure 4.

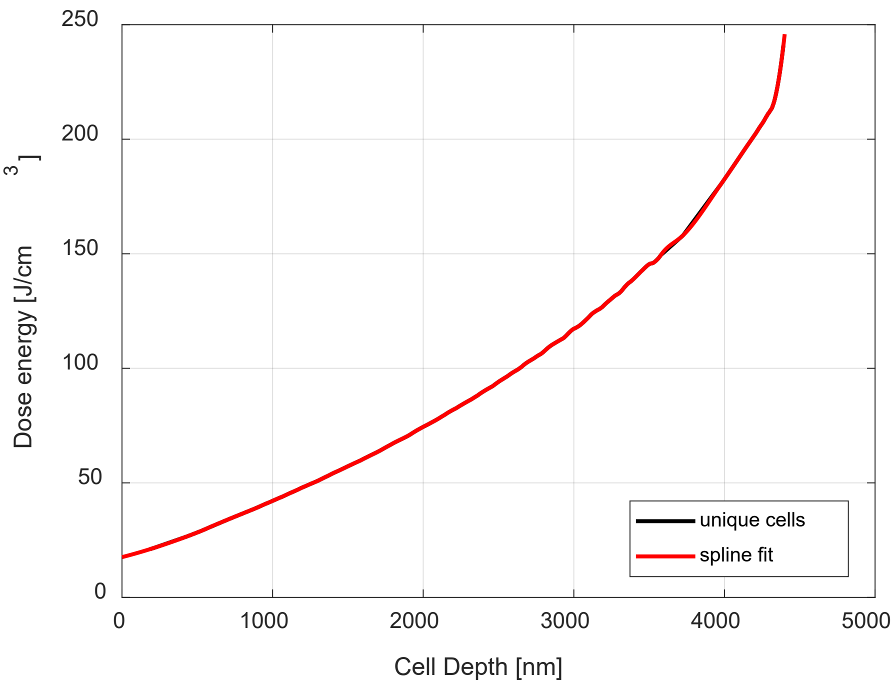

Supplementary figure 4. Graphical plot of constant dose energy (J/cm^3) and a spline fit over the data to obtain unique cell depths.

Based on this new dose energy map, we computed a new dose pattern and back substituted in equation 1 to obtain the number of scans for variable dose strategy. A plot of the constant scan and variable scan patterns were obtained as shown in supplementary figure 5.

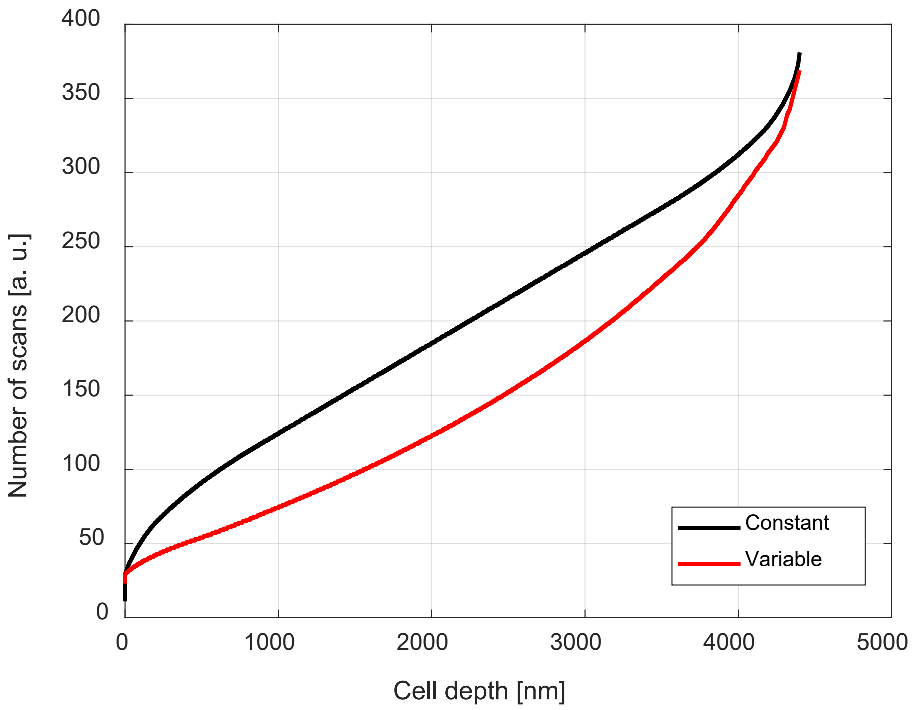

Supplementary figure 5. Graphical plot comparison between the constant and variable scan patterns in terms of the number of scans required to obtain discrete cell depths.

The computed variable dose pattern enables us to achieve a dosing strategy that ensures discrete cell depths and allow a uniform distribution of cell depth across the given cell depth range, as shown in scan maps in supplementary figure 6 and through histogram in supplementary figure 7.

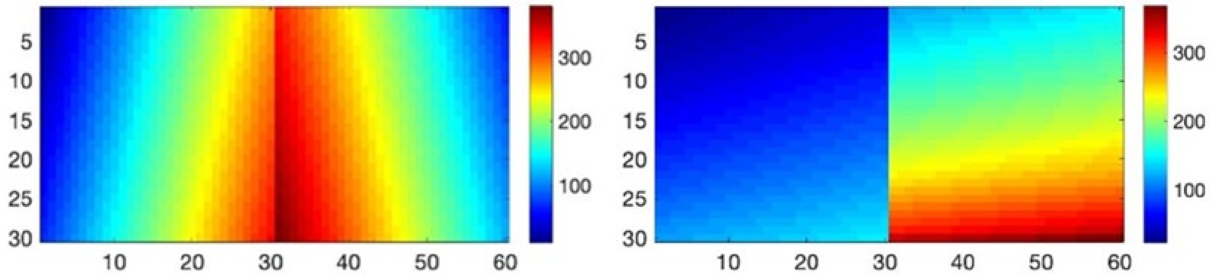

Supplementary figure 6. Comparison of color map of x-ray exposure dose across the chip in the constant (left) and variable (right) dose strategies (Red – High exposure and blue – low exposure). X and Y-axis denote the row and column numbers, respectively.

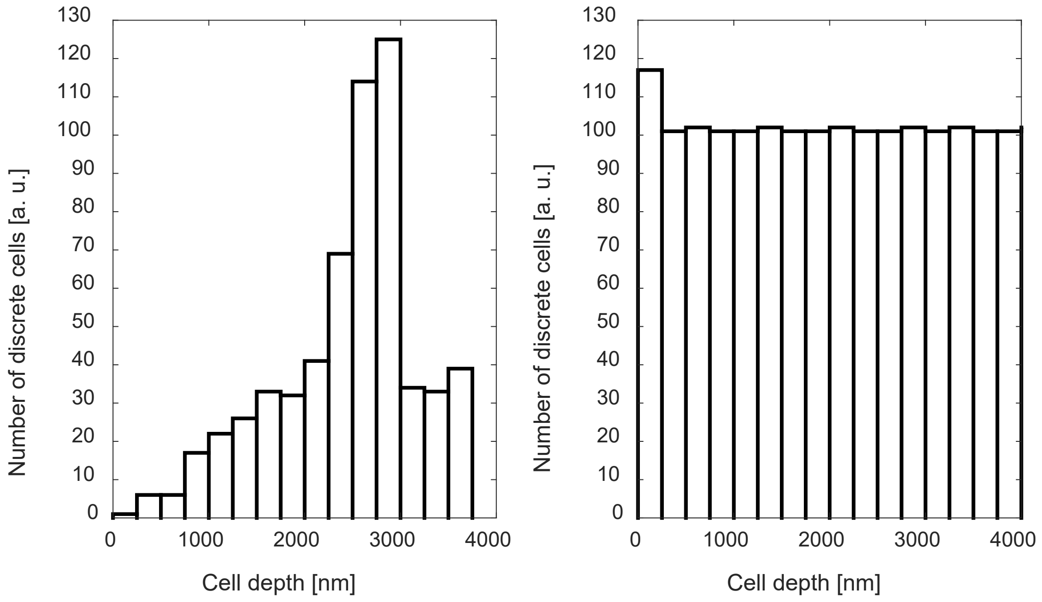

Supplementary figure 7. Histogram plot comparing the constant (left) and variable (right) dose strategies in terms of the number of discrete cells (y-axis) at different cell depth ranges (x-axis).

The minimum dose step (increment) given was 0.875 J/cm^3 and the range of doses given was in between 0.875 J/cm^3 (lowest) and 2467.5 J/cm^3 (highest). These dose ranges are typically deposited with a ring current of Synchrotron at 210 mA and with an x-ray scanner velocity of 60 mm/sec. We can further reduce the minimum dose deposited with a lower ring current or by increasing the x-ray scanner velocity and vice versa.
