# Supplementary material for: Wafer scale manufacturing of high precision micro-optical components through X-ray lithography yielding 1800 Gray Levels in a fingertip sized chip
Source: Sci Rep. 2022 Feb 17;12:2730. doi: 10.1038/s41598-022-06688-5 (PMC8854699; doi:10.1038/s41598-022-06688-5)
Supplement: Supplementary file 3 — Supplementary Information 3. [file 41598_2022_6688_MOESM3_ESM.pdf]

Statistical study on surface roughness of the 1800 gray levels

A statistical study by profilometry was carried out to analyse the surface roughness across 1800 gray level regions, which is vital for optical application. Phase-shifting interferometry was performed to measure the surface roughness of the individual cells. 1800 gray levels comprised 30 rows and 60 columns and we measured the surface roughness at equidistant cells across the entire chip containing in total roughness measurement of 98 points at 98 different cells. The surface roughness value of each cell measured is recorded in an excel chart and provided in supplementary table 2.

The following parameters were measured in each cell/gray level analysed for surface roughness.

Ra – Average roughness calculated over the entire measured array
Rq – Root mean squared roughness calculated over the whole of the measured array
Rt – Peak to valley difference calculated over the whole of the measured array

The median values, error bars, and outliers of the surface roughness values measured in 98 cells were found using the Whisker plots, as shown in supplementary figure 8. Supplementary figure 9 shows that the average roughness Ra varies between 0.21 and 1.11 nm, supplementary figure 10 shows that the root mean squared roughness Rq varies between 0.26 and 1.42 nm, and supplementary figure 11 shows that the peak to valley difference Rt varies between 1.64 and 8.14 nm.

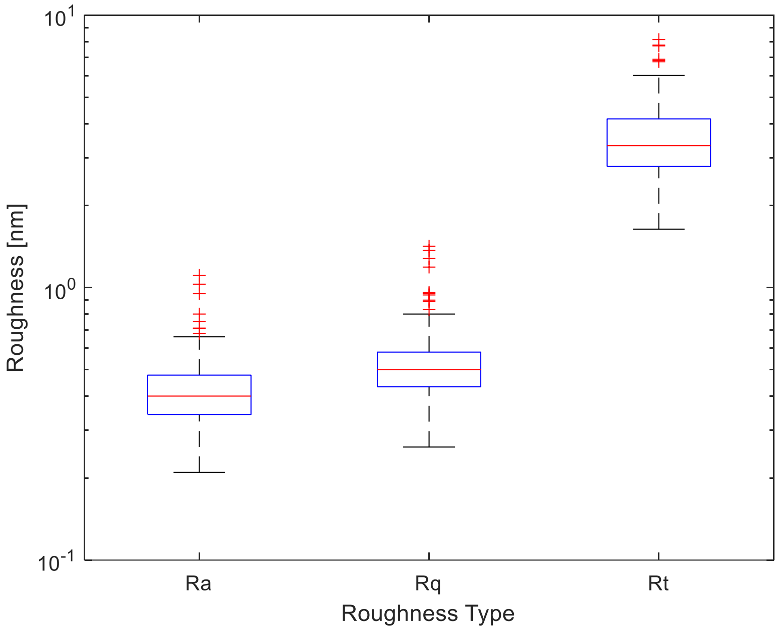

Supplementary figure 8. The Whisker plot on the surface roughness values measured in 98 cells.

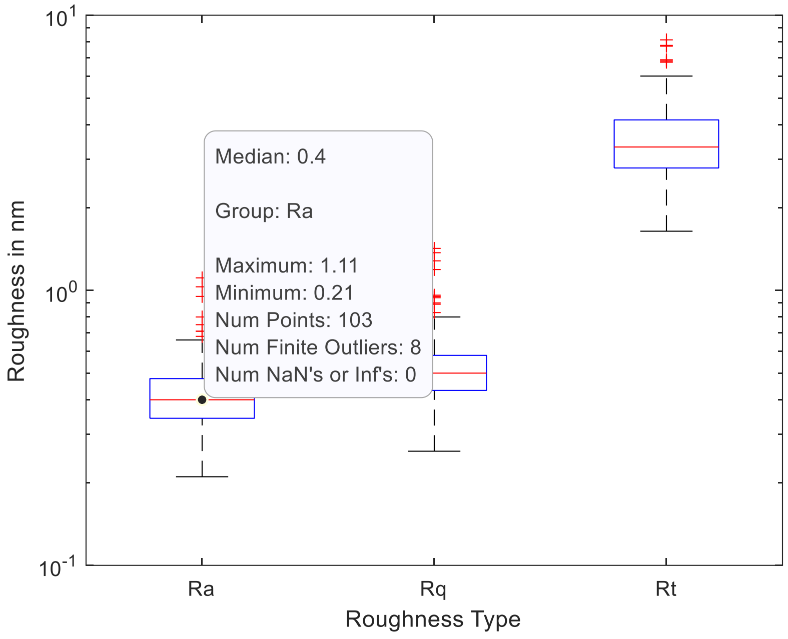

Supplementary figure 9. The Whisker plot showing the range and median values of average roughness Ra calculated across the entire measured array.

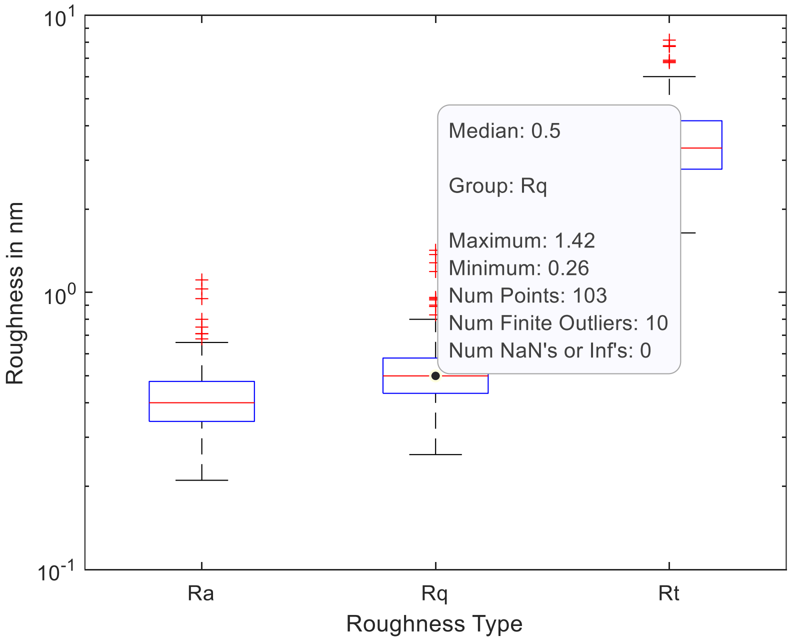

Supplementary figure 10. The Whisker plot showing the range and median values of root mean squared roughness Rq calculated across the entire measured array.

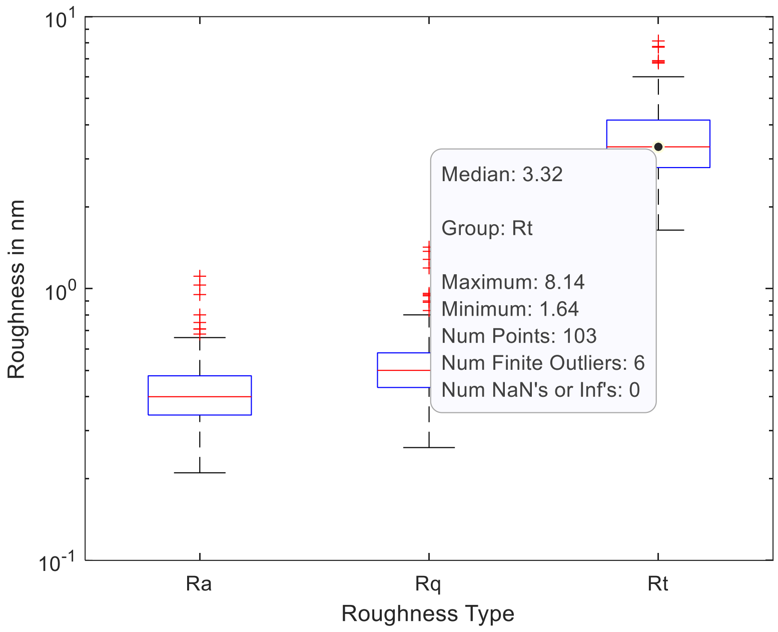

Supplementary figure 11. The Whisker plot showing the range and median values of peak to valley difference Rt calculated across the entire measured array.
