# Supplementary material for: Wafer scale manufacturing of high precision micro-optical components through X-ray lithography yielding 1800 Gray Levels in a fingertip sized chip
Source: Sci Rep. 2022 Feb 17;12:2730. doi: 10.1038/s41598-022-06688-5 (PMC8854699; doi:10.1038/s41598-022-06688-5)
Supplement: Supplementary file 4 — Supplementary Information 4. [file 41598_2022_6688_MOESM4_ESM.pdf]

Supplementary table 2. Surface roughness values measured at equidistant cells across the entire chip with 1800 gray levels.

		Ra (nm)	Rq (nm)	Rt (nm)
**Left matrix Row 1**				
	**Column 1**	0.36	0.44	2.66
	**Column 5**	0.26	0.32	2.21
	**Column 9**	0.45	0.58	4.15
	**Column 13**	0.52	0.68	5.36
	**Column 17**	0.44	0.54	3.47
	**Column 21**	0.52	0.65	4.32
	**Column 25**	0.6	0.75	5.36
**Left matrix Row 5**				
	**Column 1**	0.31	0.42	3.52
	**Column 5**	0.33	0.41	2.49
	**Column 9**	0.33	0.42	2.83
	**Column 13**	0.44	0.54	3.05
	**Column 17**	0.41	0.51	3.08
	**Column 21**	0.54	0.68	4.2
	**Column 25**	0.8	0.96	5.21
**Left matrix Row 9**				
	**Column 1**	0.32	0.41	2.43
	**Column 5**	0.42	0.52	3.12
	**Column 9**	0.54	0.67	3.64
	**Column 13**	0.32	0.41	1.99
	**Column 17**	0.36	0.45	2.65
	**Column 21**	0.39	0.48	3.12
	**Column 25**	0.5	0.64	4.55
**Left matrix Row 13**				
	**Column 1**	0.41	0.5	3.29
	**Column 5**	0.37	0.46	2.84
	**Column 9**	0.37	0.53	5.74
	**Column 13**	0.43	0.54	3.59
	**Column 17**	0.41	0.51	3.12
	**Column 21**	0.39	0.49	3.31
	**Column 25**	0.66	0.83	6.01
**Left matrix Row 17**				
	**Column 1**	0.25	0.31	1.75
	**Column 5**	0.26	0.32	2.28
	**Column 9**	0.71	0.9	5.33
	**Column 13**	0.34	0.42	2.55
	**Column 17**	0.3	0.39	2.54
	**Column 21**	0.31	0.39	2.4
	**Column 25**	0.32	0.4	2.46
**Left matrix Row 21**				
	**Column 1**	0.25	0.32	2.05
	**Column 5**	0.31	0.39	2.39
	**Column 9**	0.34	0.43	2.94
	**Column 13**	0.36	0.45	3.02
	**Column 17**	0.37	0.45	2.91
	**Column 21**	0.39	0.48	2.24
	**Column 25**	0.44	0.56	3.94
**Left matrix Row 25**				
	**Column 1**	0.45	0.55	3.32
	**Column 5**	0.35	0.44	3.19
	**Column 9**	0.47	0.58	3.47
	**Column 13**	0.57	0.7	4.31
	**Column 17**	0.43	0.54	3.33
	**Column 21**	0.4	0.5	3.26
	**Column 25**	0.21	0.26	1.64
**Right matrix Row 29**				
	**Column 1**	0.51	0.63	4.29
	**Column 5**	0.71	0.89	6.89
	**Column 9**	0.42	0.53	3.58
	**Column 13**	0.39	0.49	3.13
	**Column 17**	0.41	0.5	2.77
	**Column 21**	0.4	0.5	3.42
	**Column 25**	0.33	0.42	3.42
**Right matrix Row 33**				
	**Column 1**	0.54	0.69	4.91
	**Column 5**	0.75	0.95	6.75
	**Column 9**	0.46	0.56	3.92
	**Column 13**	0.36	0.46	3.17
	**Column 17**	0.41	0.52	3.65
	**Column 21**	0.4	0.51	4.17
	**Column 25**	0.28	0.36	3.01
**Right matrix Row 37**				
	**Column 1**	1.11	1.37	7.77
	**Column 5**	0.41	0.52	3.73
	**Column 9**	0.55	0.69	4.71
	**Column 13**	0.39	0.48	3.48
	**Column 17**	0.39	0.48	3.15
	**Column 21**	0.37	0.48	3.54
	**Column 25**	0.32	0.42	1.82
**Right matrix Row 41**				
	**Column 1**	0.56	0.68	4.12
	**Column 5**	0.41	0.5	3.12
	**Column 9**	0.63	0.8	5.1
	**Column 13**	0.56	0.7	4.92

	Column 17	0.41	0.5	3.15
	Column 21	0.44	0.56	3.36
	Column 25	0.48	0.58	1.82
Right matrix Row 45				
	Column 1	1.03	1.28	7.72
	Column 5	0.49	0.61	4.44
	Column 9	0.61	0.78	5.37
	Column 13	0.4	0.5	3.25
	Column 17	0.46	0.57	3.57
	Column 21	0.95	1.19	6.82
	Column 25	0.27	0.34	2.5
Right matrix R49				
	Column 1	0.24	0.3	1.97
	Column 5	0.68	0.94	8.14
	Column 9	0.65	1.42	4.29
	Column 13	0.34	0.43	2.7
	Column 17	0.4	0.49	3.04
	Column 21	0.39	0.5	4.79
	Column 25	0.28	0.34	2.19
Right matrix R54				
	Column 1	0.41	0.51	3.33
	Column 5	0.38	0.47	3.21
	Column 9	0.38	0.48	3.14
	Column 13	0.38	0.47	3.47
	Column 17	0.39	0.49	3.53
	Column 21	0.35	0.44	2.68
	Column 25	0.3	0.38	1.75
