# Supplementary figures and images for: Wafer scale manufacturing of high precision micro-optical components through X-ray lithography yielding 1800 Gray Levels in a fingertip sized chip
Source: Sci Rep. 2022 Feb 17;12:2730. doi: 10.1038/s41598-022-06688-5 (PMC8854699; doi:10.1038/s41598-022-06688-5)

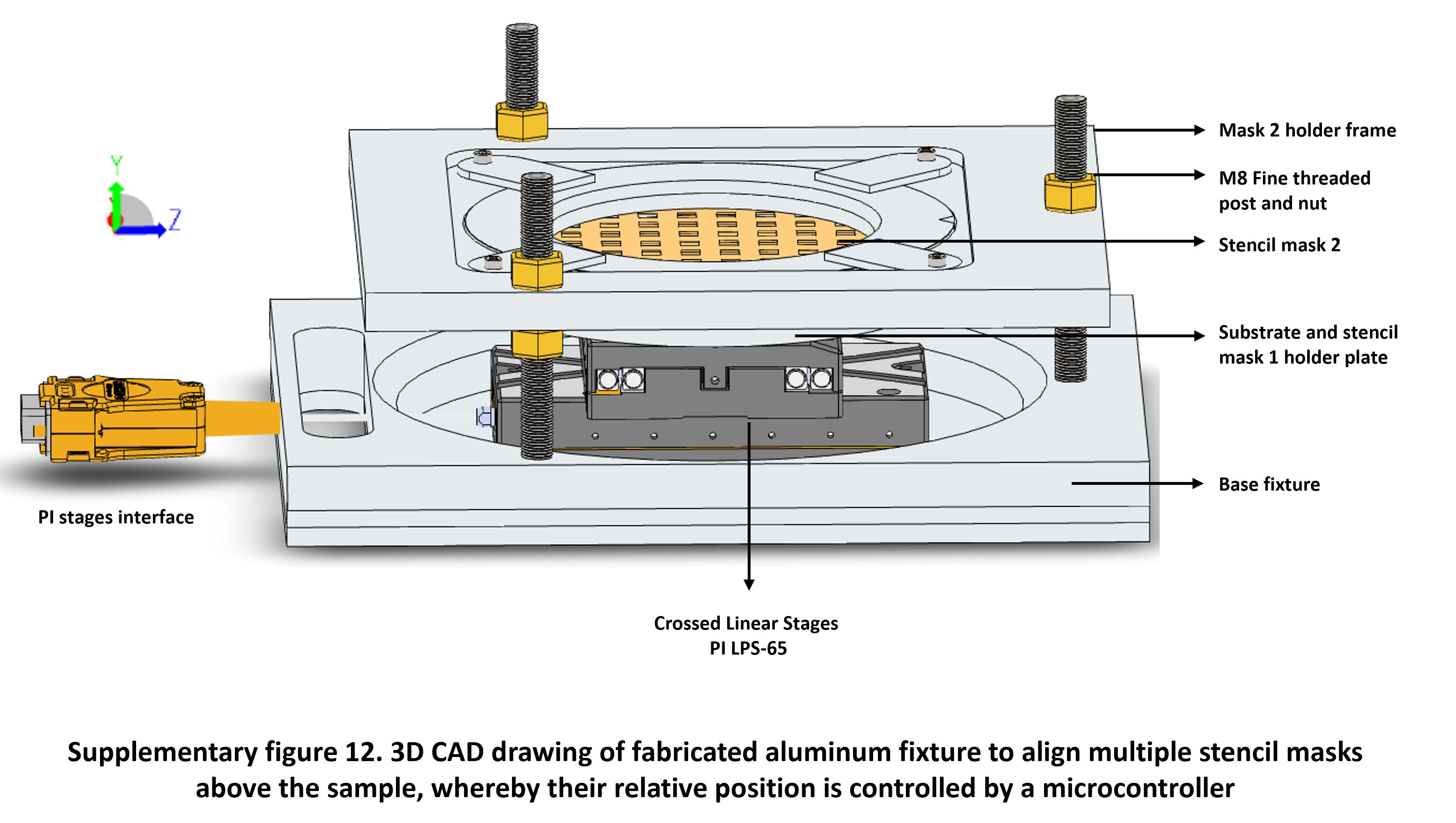

Supplement: Supplementary file 5 — Supplementary Information 5. [file 41598_2022_6688_MOESM5_ESM.tif]
